# IL-10 (−1082 G/A) polymorphism in Bataknese with schizophrenia

**DOI:** 10.1016/j.jtumed.2023.08.011

**Published:** 2023-09-11

**Authors:** Sarah A. Mardhiyah, Elmeida Effendy, Nazli M. Nasution

**Affiliations:** a Psychiatry Residency Program, Faculty of Medicine, Universitas Sumatera Utara, Indonesia; b Department of Psychiatry, Faculty of Medicine, Universitas Sumatera Utara, Indonesia; c Department of Psychiatry, Faculty of Medicine, Universitas Sumatera Utara, Indonesia

**Keywords:** تعدد الأشكال الجينية, إنترلوكين 10, علم المناعة العصبية, الفصام, باتاك, Batak, Genetic polymorphisms, Interleukin-10, Neuroimmunology, Schizophrenia

## Abstract

**Objectives:**

Three biallelic polymorphisms at the promoter region of the interleukin-10 (IL-10) gene have been associated with susceptibility to schizophrenia. The aim of this case-control study was to investigate the association between IL-10 (−1082) G/A gene polymorphisms and schizophrenia among Bataknese, a native tribe inhabiting the North Sumatera province in Indonesia.

**Methods:**

A total of 194 unrelated participants (n = 97 for each case and control groups) participated in this study. Polymerase chain reaction restriction fragment length polymorphism molecular genotyping was conducted to assess the genotype and allele distribution of IL-10 (−1082 G/A).

**Results:**

Allele variations indicated that the dominant allele in the Batak tribe was allele A, whereas homozygous GG genotypes were not found in either group. The A allele and AA genotype were found to be risk factors for developing schizophrenia (OR = 2.26, 95% CI = 1.1825–4.3559 and OR = 2.56, 95% CI = 1.280–5.152, respectively).

**Conclusion:**

Only the A allele and AA genotype of the IL-10 gene polymorphism at −1082 G/A contribute to schizophrenia susceptibility in Bataknese.

## الملخص

**أهداف البحث:**

ارتبطت ثلاثة أشكال متعددة الأشكال في المنطقة المحفزة لجين إنترلوكين-10 بقابلية الإصابة بالفصام. كان الهدف من دراسة الحالات والشواهد هذه هو دراسة العلاقة بين تعدد أشكال جينات إنترلوكين-10 عند (-1082 جم/أ) والفصام بين الباتاكنيز، وهي قبيلة من السكان الأصليين تسكن مقاطعة سومطرة الشمالية.

**طريقة البحث:**

شارك في هذه الدراسة ما مجموعه 194 شخصا غير ذي صلة (ن = 97 لكل مجموعة حالات وضوابط). تم إجراء التنميط الجيني الجزيئي باستخدام تعدد أشكال طول الجزء المقيد بتفاعل البوليميراز لتقييم النمط الجيني وتوزيع الأليل للإنترلوكين-10 (-1082 جم/أ).

**النتائج:**

أظهر تحديد اختلافات الأليل أن الأليل السائد في قبيلة باتاك هو الأليل ولم يتم العثور على الأنماط الجينية المتماثلة "جي جي" في أي من المجموعتين. يعمل الأليل والنمط الجيني "أ أ" كعامل خطر مساهم في الإصابة بالفصام.

**الاستنتاجات:**

فقط النمط الجيني للأليل و "أ أ" من تعدد أشكال جين إنترلوكين -10 عند (-1082 جم/أ) يسهم في قابلية الإصابة بالفصام في الباتاكنيز.

## Introduction

Schizophrenia, a multifaceted disorder characterized by persistent psychosis and gradual cognitive decline, affects more than 20 million individuals worldwide.^1^^,^^2^ A major hypothesis is that dopamine is associated with schizophrenia, yet current studies have indicated that schizophrenia is also associated with immune dysregulation in the central nervous system.^3^ This immune regulation is performed by microglia and astrocytes in the central nervous system, and is mediated by numerous factors, including cytokines. Interleukin-10 (IL-10) is a potent anti-inflammatory cytokine secreted by activated T-regulatory cells, Th2 cells, and B-lymphocytes. It is also known as a cytokine synthesis inhibitor factor, because it inhibits the secretion of pro-inflammatory cytokines.^4^ Interindividual differences in IL-10 secretion are associated with single nucleotide polymorphisms in the IL-10 gene. This gene is located on human chromosome 1 between 1q31and 1q32.^5^ The IL-10 gene consists of five exons and four introns, and encodes approximately 160 amino acids. Three biallelic polymorphisms have been identified in the promoter region of the IL-10 gene (−1082 G/A, −819T/C, and −592 C/A) and are believed to be associated with the level of IL-10 secretion. Dysregulated IL-10 secretion is believed to disrupt the immune response equilibrium and to underlie schizophrenia development.^6^^,^^7^ Studies examining the association between IL-10 polymorphism and schizophrenia have yielded inconsistent results,^8^, ^9^, ^10^ and studies on IL-10 polymorphism in Indonesians, particularly in the North Sumatera province, remain limited. An IL-10 polymorphism study has been conducted in individuals with periodontitis in Indonesia,^11^ yet no IL-10 polymorphism study has been performed in individuals with schizophrenia. Therefore, our study is, to our knowledge, the first to investigate IL-10 polymorphism especially in Bataknese. The Batak are the native tribe inhabiting North Sumatera, with a population of 6 million people, accounting for nearly half the population in the province. The National Health Survey of the Republic of Indonesia (*Riskesdas*, 2018) has indicated that the prevalence of schizophrenia and/or other psychotic disorders is approximately 6.9%.^12^ Data from a provincial mental hospital have indicated that, among 400 patients with schizophrenia, 60.5% (n = 242) were Bataknese.^13^ Bataknese consistently show patrilineal characteristics: ancestry is maintained through the male lineage and can be easily determined from the family name (*marga*). To maintain the purity of the lineage, most Bataknese follow the tradition of marrying those from other Batak lineages.^14^ Previous studies have reported that IL-10 polymorphism at position −1082G is strongly associated with schizophrenia and have demonstrated interethnic differences.^15^^,^^16^ Therefore, in this study, we aimed to investigate IL-10 gene polymorphism, specifically, at −1082 G/A, and associations with schizophrenia susceptibility in Bataknese.

## Materials and Methods

### Study design

This observational study was a comparative case control study. The case group consisted of Bataknese with schizophrenia, whereas the control group consisted of healthy Bataknese.

### Subjects

Eligible participants were Bataknese individuals, recognized by their *marga* (family name), with at least two first degree relatives confirmed to be Bataknese through the paternal lineage. The case group comprised Bataknese diagnosed with schizophrenia according to the DSM V diagnostic criteria,^17^ whereas the control group comprised Bataknese without any ongoing psychiatric disorders or a history of psychiatric disorders, as determined through a Mini International Neuropsychiatry Interview structured interview.^18^ In the control group, individuals with families with psychiatric comorbidities and any people diagnosed with autoimmune disorders were also excluded from the study. A total of 194 unrelated participants (97 participants in each group) provided informed consent prior to participation. A 3 ml blood sample was withdrawn from each participant, kept in an EDTA tube, and stored in a cooler box at <4 °C before transport to the laboratory.

### DNA isolation

DNA isolation was performed according to the manual of the Wizard Genomic DNA Purification kit, Promega-A1120.^19^ Initially, blood samples were centrifuged (3000 rpm for 10 min) until a buffy coat layer was visible. Subsequently, 300 μl of buffy coat was transferred to a microcentrifuge tube, and 900 μl of erythrocyte lysis buffer was added. The mixture was incubated for 5 min in a refrigerator and centrifuged until supernatant was formed and could be separated. Nuclear lysis solution (300 μl) and protein precipitation solution (100 μl) were added and centrifuged (13,000 rpm for 3 min) until a supernatant formed and could be separated from the mixture. The supernatant was placed in a new microcentrifuge tube, 300 μl isopropanol was added, and centrifugation was performed at 13,000 rpm for 1 min. The DNA pellet that formed at this stage was washed with 300 μl 70% ethanol. Finally, 100 μl DNA rehydration solution was added to the pellet and stored for long-term use at −20 °C.

### Polymorphism of IL-10 (−1082 G/A) identification

Polymerase chain reaction-restriction fragment length polymorphism techniques were as described by Ozbey et al.^20^ The PCR denaturation cycle started at 95 °C for 5 min, and was followed by another 35 cycles of denaturation at 94 °C for 30 s, annealing at 55 °C for 30 s, and extension at 72 °C for 30 s by using Gradient PC (Applied Biosystems). PCR products were stored at 37 °C for 24 h before the *MnlI* (Thermo Scientific, USA) restriction enzyme was added and incubated at 37 °C for 2 h. Genotypes were identified through 3% agarose gel electrophoresis.

### Statistical analysis

The differences in genotype and allele frequencies between the case and control groups were analysed with chi square test, and *p* ≤ 0.05 was considered significant. The strength of the association was expressed as the odds ratio (OR), in which an OR < 1 indicates a negative association or protective factor; an OR > 1 indicates a contributing risk factor; and an OR = 1 indicates no association of the disease with respect to a particular genotype or allele variation.^21^

## Results

The demographic characteristics of participants in this study are summarized in Table 1. Men predominated in the case group (*p* = 0.008). Only 10.3% (n = 10) of Bataknese with schizophrenia were employed at the time of the study, and only 3.1% (n = 3) of these individuals had been able to complete a diploma/university degree.

**Table 1:** Demographic characteristics of participants.

Characteristics	Groups		
	Case (n = 97)	Control (n = 97)	*p*
Gender			
Male	81 (83.5%)	64 (66.0%)	0.008∗
Female	16 (16.5%)	33 (24.5%)	
Employment			
Yes	10 (10.3%)	89 (93.7%)	0.001∗
No	87 (89.7%)	6 (6.3%)	
Education			
Primary	12 (12.4%)	0 (0.0%)	0.001∗∗
Highschool	82 (84.5%)	47 (48.5%)	
Diploma/university	3 (3.1%)	50 (51.5%)	
Age (years)	34.27 ± 6.85	31.01 ± 7.31	
Illness duration	11.31 ± 5.24	–	
Onset	23 (16–37)	–	

∗ Chi- square, ∗∗ Mann-Whitney.

Statistical analysis indicated significant differences in employment and education status between groups (*p* = 0.001). The mean age of Bataknese with schizophrenia in this study was 34.27 ± 6.85; these participants had lived with schizophrenia for 11.31 ± 5.24 years and had median age of onset of 23 years.

**Figure 1:**
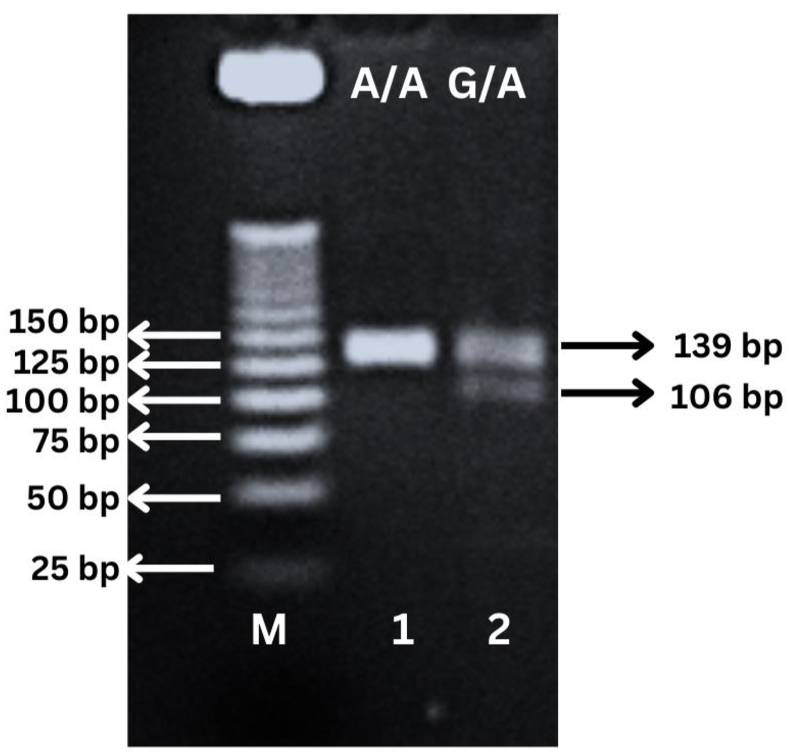
Representative gel electrophoresis showing the PCR products of IL-10 polymorphism at −1082 G/A using 25 bp ladder; M is molecular marker, lane 1 represents homozygous AA subject, and lane 2 represents heterozygous GA subjects. G allele cuts with restriction enzyme *MnI*I, generating 106 bp (lane 2), while A allele does not cut (lane 1). Homozygote GG subject was not found in any subjects in this study.

Figure 1 shows gel electrophoresis with a 25 bp ladder. The genotypes of IL-10 (−1082) genotypes were named according to the presence or absence of enzyme restriction sites. Homozygous AA was represented by an absence of restriction sites, and thus the PCR products remained uncut (139 bp). Homozygous GG was represented by the presence of restriction sites at 106 and 33 bp, but was not found in any participants. Heterozygous G/A was represented by the presence of restriction sites at 139, 106, and 33 bp.

Table 2 shows a cross tabulation of genotype and allele variation in IL-10 (−1082 G/A). The dominant allele among Bataknese was the A allele, and the homozygous GG genotype was not found. The frequency of the AA genotype was significantly higher (*p* = 0.011) among Bataknese with schizophrenia (84.5%) than in the control group (68%). The frequency of the A allele was also significantly higher (*p* = 0.018) among Bataknese with schizophrenia (92.3%) than in the control group (84%). These results indicated that the A allele and AA genotype are risk factors for schizophrenia development in Bataknese (OR = 2.26, 95% CI = 1.1825–4.3559 and OR = 2.56, 95% CI = 1.280–5.152, respectively).

**Table 2:** Cross tabulation of allele and genotype variations of IL-10 (−1082 G/A).

Variations	Groups			OR (95% CI)
	Case	Control	*p*	
G allele	15 (8.4%)	31 (19.0%)	0.018	2.26 (1.1825–4.3559)
A allele	179 (91.6%)	163 (81.0%)		
GG	15 (15.5%)	31 (32%)	0.011	2.56 (1.280–5.152)
AA	82 (84.5%)	66 (68%)		

## Discussion

Schizophrenia is a complex psychiatric disorder affecting various social aspects of life. In this present study, schizophrenia was more prevalent in men, as also described in a cohort study by Sanchez et al.^22^ involving more than 3 million participants aged 15–64 years old in SpainThe study showed that the prevalence of schizophrenia in men was 64.3%, a percentage approximately twice that in women (35.7%). Across all age groups, schizophrenia predominated in men. This finding was supported by a meta-analysis indicating sex-specific differences in schizophrenia incidence, with men having greater risk than women (RR = 1.42, 95% CI: 1.30–1.56).^23^ Our study findings contrasted with those from other studies suggesting that the morbidity risk of schizophrenia is 1%, and that no difference in prevalence exists between sexes, although the possibility of schizophrenia incidence is indeed higher among men.^24^^,^^25^ Additional factors that may have roles in this sex-specific difference are access to mental health services and help-seeking behaviour, which may differ between men and women, thereby affecting the prevalence rate of schizophrenia by sex. A study in China has found a higher prevalence of schizophrenia in women than men, because mental health services are more stigmatized for women than men in China, thus resulting in a smaller proportion of women than men with psychosis being treated appropriately and increasing the observed prevalence of schizophrenia among women.^26^ The onset of schizophrenia generally occurs at the age of 21–25 years, but is generally 3–5 years later in women than in men. This demographic feature was also observed in this present study. The onset of schizophrenia at 40–50 years of age, although rare, is also more common in women (66%–87%) than men.^27^^,^^28^ The mean age of participants in our study was similar to that in other studies. Schizophrenia is most commonly found at the age of 30–40 years, and its prevalence decreases in older age groups.^29^ Even studies conducted in communities with high incomes have indicated that individuals with schizophrenia gradually lose productivity and later become unable to retain jobs or continue academic study.^19^^,^^30^^,^^31^ Studies have also indicated that 98% of individuals with schizophrenia exhibit lower performance than healthy individuals. Diminished cognitive function in schizophrenia is associated with a decrease in the thickness of the frontal-temporal cortex area, independently of the severity of psychotic symptoms in schizophrenia. This is associated with a diminished ability to pay attention and learn, thus resulting in a decline in personal and social functioning.^32^^,^^33^

In this present study, individuals with the A allele and AA genotype were more susceptible to schizophrenia development, similarly to the findings from a study by Almoguera et al. involving 241 White people with schizophrenia.^34^ We also did not identify the GG genotype, similarly to a study of IL-10 polymorphism among Javanese with periodontitis in Indonesia. That study also found that the A allele was dominant and did not the identify the homozygous GG genotype.^11^ Another IL-10 polymorphism study in a Turkish population has reported frequencies of the GG genotype in schizophrenia and control groups of 0% and 0.3% respectively; however, polymorphism at position −1082G/A did not differ between the case and control groups.^20^ A low GG genotype frequency has also been reported in another study, in which the GG genotype was significantly lower among people with schizophrenia (2.21%) than controls (7.53%). Regarding the low frequency of the GG genotype, genetic drift, an evolutionarily process resulting in random fluctuations of allele frequencies among generations, may possibly provide an explanation. Genetic drift may cause genetic variations to disappear, thus resulting in the disappearance certain gene variants, or increased frequency or even fixation of previously rare alleles. Major effects can result when a population is extremely decreased, e.g., because of natural disasters (bottleneck effect) or mass migration events in which a population splits to create new colony.^35^^,^^36^

Our results are supported by those from a study from Al Amsary^9^ in a Saudi population, in which IL-10 (−1082 G/A) was found to be associated with schizophrenia. However, that study has indicated that, instead of being risk factors, the AA and GG genotypes are considered protective. Rajasekaran^37^ reported no association of IL-10 (−1082 G/A) variants with schizophrenia, but because the control group did not exhibit Hardy–Weinberg equilibrium (*p* = 0.02), the results are inconclusive. A study in Poland also yielded different results, but that study examined only people with paranoid schizophrenia. Other studies have reported conflicting results, stating that IL-10 (−1082 G/A) is not associated with schizophrenia, in which the study population was different from that in our study^10^^,^^38^ thus indicating that population selection regarding ethnicity might have influenced the differences across several studies.

Decreased IL-10 serum levels are considered an underlying mechanism in schizophrenia, because IL-10 participates in the anti-inflammatory response. A study by Xiu et al. has shown that IL-10 serum levels are markedly lower in drug-naïve people with schizophrenia.^39^ Although serum IL-10 levels are elevated, immunosuppressive effects might compensate for prior elevations in pro-inflammatory cytokines in people with schizophrenia.^40^ Nevertheless, a potential limitation in our study is that we did not examine the association between polymorphism and IL-10 serum levels in our participants. We based our hypothesis on findings from an in vitro study by Turner et al. in peripheral leukocytes. That study has indicated that the A allele at position −1082, compared with the than G allele, is associated with lower IL-10 secretion. The IL-10 gene polymorphism involving a guanine (G) base substitution to adenine (A) at position −1082 is known to be associated with specific promoter regions' transcriptional activity and recognition site, thus probably decreasing transcription rates and consequently IL-10 serum levels.^41^

Genetic association studies may often yield inconsistent results, possibly because of selection bias, e.g., where the study is conducted or the population in which the study is performed. Population structure may also contribute to biased results, owing to genetic mixing or cryptic relatedness, in which individuals share a common ancestry that is unknown to the investigators; therefore, genetic association studies ideally should be carried out in cohorts with the same ethnicity or population structure.^42^

## Conclusion

Our results suggested that the A allele and AA genotype at the −1082 position are risk factors for developing schizophrenia. To our knowledge, this is the first study investigating IL-10 polymorphism in the Bataknese population. However further studies are encouraged to examine IL-10 polymorphisms at other positions and their associations with IL-10 serum levels.

## Source of funding

This research did not receive any specific grant from funding agencies in the public, commercial, or not-for-profit sectors.

## Conflict of interest

The authors have no conflict of interest to declare.

## Ethical approval

Before beginning this study, we obtained approval, in accordance with Declaration of Helsinki and Nuremberg Code, from the Ethical Committee of Health Research of Universitas Sumatera Utara (letter number 254/KEPK/USU/2022).

## Consent

Informed consent forms clearly explaining the study procedures, risks, and code of conduct in cases of any emergencies (e.g., bleeding), were given to all participants. All participants' personal information was kept confidential and was only fairly used in the study.

## Authors contributions

SAM, the corresponding author, conceived and designed the study and was a major contributor in conducting the research, collecting and organizing the data, and writing the original and revised drafts. Initial conceptualisation, methods, and validation were conducted by EE and NMN. NMN also substantially contributed to the methods and interpretation in statistical analysis of the data in the study. All authors have critically reviewed and approved the final draft and are responsible for the content and similarity index of the manuscript. All authors have also read and approved the final manuscript.

## Acknowledgment

We thank Lavarina Winda and Hidayat for assisting in molecular phenotyping throughout this study.

## Data availability statement

Data and materials in this study can be requested upon reasonable request directly from the corresponding author.
